# The Current State of Treatment and Future Directions in Cutaneous Malignant Melanoma

**DOI:** 10.3390/biomedicines10040822

**Published:** 2022-03-31

**Authors:** Madison Ernst, Alessio Giubellino

**Affiliations:** Department of Laboratory Medicine and Pathology, University of Minnesota, Minneapolis, MN 55455, USA; ernst215@umn.edu

**Keywords:** metastatic melanoma, targeted therapy, immunotherapy, combined therapies, tumor relapse, tumor resistance

## Abstract

Malignant melanoma is the leading cause of death among cutaneous malignancies. While its incidence is increasing, the most recent cancer statistics show a small but clear decrease in mortality rate. This trend reflects the introduction of novel and more effective therapeutic regimens, including the two cornerstones of melanoma therapy: immunotherapies and targeted therapies. Immunotherapies exploit the highly immunogenic nature of melanoma by modulating and priming the patient’s own immune system to attack the tumor. Treatments combining immunotherapies with targeted therapies, which disable the carcinogenic products of mutated cancer cells, have further increased treatment efficacy and durability. Toxicity and resistance, however, remain critical challenges to the field. The present review summarizes past treatments and novel therapeutic interventions and discusses current clinical trials and future directions.

## 1. Introduction

Invasive melanoma accounts for only 1% of all skin cancers, yet is the leading cause of skin-cancer-related deaths. In 2022, an estimated 99,780 new cases of invasive melanoma will be diagnosed, and an estimated 7650 people will die from the disease in the United States, making it the fifth most common malignancy in both sexes [1]. While the incidence of melanoma continues to increase from prior years, mortality rates have declined by ~4% per year since 2015 [1]. This decline reflects significant advancements in treatment for advanced and metastatic (mM) disease, the most significant of which are targeted therapies and immunotherapies. Unlike chemotherapies or radiation, in which the therapy directly induces cancer cell death, immunotherapies stimulate the patient’s immune system to control and eliminate the tumor. The high immunogenicity and somatic mutation burden of melanoma likely contribute to the success of immunotherapy [2]. Advantages of immunotherapies over traditional cancer treatments include increased durability for long-term control or even cure and more precisely targeted antitumor activity that spares healthy tissues, many times with comparable or even reduced overall toxicity. A better understanding of the molecular landscape of melanoma has helped to refine not only our understanding of the disease but also to propose novel targets or target combinations for selective therapeutic intervention. As technologies around genetic sequencing, modification, and bioinformatics become more advanced and accessible, immunotherapy has the potential to become even more effective with personalized agents and regimens.

Currently, there are three types of immunotherapies currently approved by the U.S. Food and Drug Administration (FDA) for the treatment of advanced melanoma: (1) T-cell-stimulating cytokines (i.e., interferon (IFN)-α2b and interleukin-2 (IL-2)); (2) T-cell exhaustion-mitigating immune-checkpoint inhibitors (ICI); and (3) a dendritic cell (DC)-activating oncolytic virus (T-VEC). Still others, such as adoptive cell transfer (ACT), hold strong promise for the future.

## 2. Early Therapies for Malignant Melanoma

### 2.1. Chemotherapy

The first pharmacologic treatment option for advanced melanoma was chemotherapy. Single agents, such as dacarbazine and temozolomide, had partial response rates of 15–28%, with minimal potential for a complete response. Combining multiple chemotherapeutics, such as in the CVD (cisplatin, vinblastine or vindesine, and dacarbazine) and Dartmouth (carmustine, dacarbazine, cisplatin, and tamoxifen) regimens, improves clinical response rates but increases toxicity, and does not improve overall survival (OS) [3,4]. With chemotherapy alone, the median overall survival for patients with advanced melanoma is 5.1 months from diagnosis with a 3-year survival probability of less than 5% [5]. While chemotherapy has since been largely replaced by new therapies that provide survival benefit, systemic and localized (i.e., isolated limb perfusion and isolated limb infusion) therapy with chemotherapeutic agents remains a viable palliative option for some patients with refractory, progressive, relapsed, or unresectable melanomas [6,7,8,9,10,11].

### 2.2. Interferon-α2b

Interferon (IFN)-α2b is a recombinant form of human IFN-α with antiviral and antitumor properties. It was the first immunotherapy approved for melanoma, first as an adjuvant treatment in 1996 and then as first-line therapy in 1998. By binding to IFN receptors 1 and 2, the drug triggers multiple dose- and time-dependent immunostimulatory effects, including upregulation of major histocompatibility complex 1 (MHC1) on tumor cells, enhanced activation of antitumor cytotoxic T lymphocytes (CTLs), depression of T regulatory cells (Tregs), enhanced dendritic cell (DC) response, and decreased intercellular adhesion molecule (ICAM) expression [12,13,14]. In 1996, high-dose (HD) IFN-α2b became the first adjuvant therapy, approved for use in stage IIB and III melanoma patients following surgical resection (Table 1). Initial trials demonstrated significantly improved 5-year relapse-free survival (RFS) (37% vs. 26%) and OS (46% vs. 37%) compared to observation alone [15]. Twelve years of longitudinal results from a pooled analysis of over 2000 patients verified the relapse-free survival (RFS) benefit, but failed to demonstrate improved OS [16]. Multiple meta-analyses had similar findings [17,18,19]. Furthermore, HD IFN-α2b treatment is limited by high toxicity, with studies reporting dose reductions in 28–55% of patients and toxicity attrition rates of 10–26% [20]. Peginterferon-α2b (Peg-IFN), which has a longer half-life than IFN-α2b, was approved for adjuvant use in 2011 after demonstrating significant improvement in 7-year RFS compared to observation (39.1% vs. 34.6%); however, like HD IFN-α2b, it could not provide an OS benefit [21,22]. HD IFN-α2b remained the standard adjuvant therapy for high-risk melanoma until ipilimumab’s approval in 2015.

Low-dose (LD) IFN-α2b was approved as a first-line therapy for stage II melanoma patients in 1998 based on a trial showing improved 5-year RFS (43% vs. 51%) and a trend toward improved OS (24% vs. 32%) compared to observation [23]. It does not have a significant clinical benefit in mM [24].

Today, IFN-α is no longer first-line agent for most patients; however, it may still have utility as an auxiliary immunostimulatory agent, enhancing the clinical benefits of other immunotherapies. Ongoing investigations using IFN-α2b include combination therapy with radiation and autologous dendritic cell vaccines for unresectable or treatment-resistant advanced melanoma (NCT01973322), in combination therapy with tumor-infiltrating lymphocytes (TIL) and nivolumab (NCT03638375), and in comparative studies investigating relative efficacies of various adjuvant therapies (NCT03178123, NCT02506153). Each of these therapies are discussed in more detail below. The possibility of a more targeted IFN-α is also under investigation with modakafusp alfa (TAK-573), which aims to improve efficacy and reduce off-target effects and toxicity by delivering IFN-α2b directly and exclusively to CD38+ cells (i.e., effector T cells, natural killer (NK) cells, B cells) (NCT04157517).

**Table 1:** Summary of approved treatments for metastatic melanoma and corresponding clinical trials.

Trial	Agent	Population	Results	Toxicity	Survival Benefit	FDA Approval
ECOG trial EST 1684 (Kirkwood et al., 1995) [15]	Adjuvant IFN-α2b vs. placebo	High-risk, resected melanoma patients (*n* = 287)	IFNa-2b increased RFS from 1 to 1.7 years (*p* = 0.0023) and OS from 2.8 to 3.8 years (*p* = 0.02)	>80% overall; with 76% grade III/IV	No	Approval of adjuvant IFNa-2b for resected, high-risk melanoma in 1995
Multi-institution prospective trial (Atkins et al., 1999) [25]	HD IL-2 (*n* = 270)	Unresectable or mM	ORR 16%, CRR 6%, PRR 10%; median survival duration 12 months; median PFS for responding patients 13.1 months (58% at 1 year)	Grade 3/4 toxicity: >45%	NA	Approval of HD-IL2 for stage IV mM in 1998
EORTC 18991 Phase III RCT (Eggermont et al., 2008) [22]	Adjuvant PEG-IFNα2b (*n* = 627) vs. observation (*n* = 629)	Recently resected stage III melanoma	PEG-IFN had improved 4-year recurrence-free survival (45.6% vs. 38.2%; *p* = 0.01)	Grade 3/4 toxicity was increased from 12% with observation to 45% with PEG-IFN	No	PEG-IFNα2b approved for adjuvant melanoma treatment in 2011
Double-blind, phase III RTC (Hodi et al., 2010) [26]	Ipilimumab + dacarbazine (*n* = 250) vs. dacarbazine+ placebo (*n* = 252)	Previously untreated, unresectable stage III or IV mM	Ipilimumab + dacarbazine had significantly longer OS (11.2 mo vs. 9.1 mo) and 1-year survival rates (47% vs. 36%, *p* < 0.001). RRs were similar (I + D = 15.2% vs. 10.3%; *p* = 0.09).	Grade 3/4 toxicity rate significantly higher with I + D (56% vs. 27% *p* < 0.001)	Yes	Approval of ipilimumab for mM in 2011
BRIM-3 (Chapman et al., 2011) [3]	Vemurafenib (*n* = 337) vs. dacarbazine (*n* = 338)	Unresectable, previously untreated stage IIIC or IV melanoma with BRAF V600E mutation	Vemurafenib had a 63% relative reduction in risk of death and a 74% risk reduction in risk of progression or death (*p* < 0.001 for both). Vemurafenib RR 48% vs. 5% for dacarbazine.	Vemurafenib required more dose reductions (38% vs. 16%) and caused SCC in 18% vs. <1% in dacarbazine	Yes	Approval of vemurafenib for advanced melanoma in 2011
Phase III RCT (Flaherty et al., 2012) [27]	Trametinib (T) (*n* = 214) vs. chemotherapy (dacarbazine or paclitaxel) (*n* = 108)	Unresectable, BRAF V600E/K+ stage IIIC or IV cutaneous melanoma	Trametinib increased ORR (22% vs. 8%, *p* < 0.01), median PFS (4.8 mo vs. 1.5 mo; *p* < 0.001), 6-month OS (81% vs. 67%; *p* = 0.01) and decreased risk of death by 46%	Trametinib required more dose reductions 27% vs. 10%	Yes	Approval of trametinib BRAF V600E/K+ melanoma in 2013
Phase III multi-institutional RCT (Hauschild et al., 2012) [28]	Dabrafenib (*n* = 187) vs. dacarbazine (*n* = 63)	Treatment-naïve, BRAF V600E+ unresectable stage III or IV melanoma	Dabrafenib improved PFS (5.1 mo vs. 2.7 mo) and RR (50% vs. 6%)	Toxicity-related dose reductions were needed in 28% of dabrafenib and 17% of dacarbazine patients	NA (underpowered)	Approval of dabrafenib for BRAF V600E+ unresectable stage III or IV melanoma in 2013
COMBI-d phase III RCT (Robert et al., 2015) [29]	Dabrafenib + trametinib (*n* = 352) vs. vemurafenib (*n* = 352)	Treatment-naïve, BRAF V600+ advanced or unresectable stage IIIC-IV melanoma	Dabrafenib + trametinib improved 1-year OS (72% vs. 65%; *p* = 0.005), PFS (11.4 mo vs. 7.3 mo), and ORR (64% vs. 51%; *p* < 0.001)	Grade 3/4 adverse event rates: 52% with dabrafenib + trametinib vs. 63% with vemurafenib	Yes	Dabrafenib + trametinib approved for BRAF V600E/K+ unresectable or mM in 2014
CheckMate-066 double-blind phase III RCT (Robert et al., 2015) [30]	Nivolumab (*n* = 210) vs. dacarbazine (*n* = 208)	Treatment naïve, BRAF wt unresectable or mM	Nivolumab improved 1-year survival rates (72.9% vs. 42.1%, *p* < 0.001), increased PFS (5.1 mo vs. 2.2 mo, *p* = 0.001), and improved ORRs (40.0% vs. 13.9%; *p* < 0.001)	Nivolumab had reduced grade 3/4 toxicity (11.7% vs. 17.6%)	Yes	Nivolumab approved for treatment-naïve BRAF wt and treatment-resistant BRAF mu unresectable or mM in 2014
KEYNOTE-006 RCT, phase III (Robert et al., 2015) [31,32]	Pembrolizumab vs. ipilimumab	Unresectable stage III or IV BRAF mu melanoma previously treated with ≤1 systemic therapy	Pembrolizumab had increased 6-month PFS (47% vs. 26.5%, *p* < 0.001), RR (33% vs. 12%; *p* < 0.001). Relative risk of death decreased by 31% with pembrolizumab.	Grade 3–5 adverse events lower with pembrolizumab (13% vs. 20%)	Yes	Pembrolizumab granted regular approval for unresectable or mM in 2015, replacing ipilimumab as first-line treatment
CheckMate 067 double-blind, phase III RCT (Larkin et al., 2015) [33,34]	Nivolumab (*n* = 316) vs. nivolumab + ipilimumab (*n* = 314) vs. ipilimumab (*n* = 315)	Treatment-naive, unresectable stage III or IV melanoma	Nivolumab with or without ipilimumab had improved PFS (Nivo + Ipi = 11.5 mo, Nivo = 6.9 mo, Ipi = 2.9 mo) and improved ORR (N + I = 57.6%, *n* = 43.7% vs. I = 19%)	Grade 3–4 adverse event rates: I = 27.3%, *n* = 16.3%, *n* + I = 55%	Yes—both Nivo and Ipi+Nivo at 4-year follow-up study (33)	Ipilimumab + nivolumab approved for advanced melanoma in 2015
OPTiM phase III RCT (Andtbacka et al., 2015) [35]	T-VEC (*n* = 295) vs. GM-CSF (*n* = 141)	Unresectable stage IIIB–IV melanoma	T-VEC improved DRR (16.3% vs. 2.1%; *p* < 0.001) and ORR (26.4% vs. 5.7%) with slightly longer median OS (23.3 mo vs. 18.9 mo; *p* = 0.051)	>2% grade 3/4 adverse events	No	T-VEC approved for recurrent local treatment of cutaneous, subcutaneous and nodal lesions in resected melanoma in 2015
coBRIM phase III RCT (Larkin et al., 2014 and Ascierto et al., 2016) [36,37]	Vemurafenib + cobimetinib (*n* = 247) vs. vemurafenib (*n* = 248)	Treatment-naïve, BRAF-mu unresectable or mM	Vemurafenib + cobimetinib increased median PFS (12.3 mo vs. 7.2 mo; *p* < 0.001), ORR (69.6% vs. 50%), and OS (22.3 mo vs. 17.4 mo)	Cobimetinib + vemurafenib had more grade 3/4 adverse events (37% vs. 28%)	Yes	Vemurafenib + cobimetinib approved for BRAF mu unresectable or mM in 2015
COMBI-AD double-blind phase III RCT (Long et al., 2017 [38] and Dummer et al., 2020 [39])	Adjuvant dabrafenib + trametinib (*n* = 438) vs. placebo (*n* = 432)	Resected stage III melanoma with BRAF V600E/K mutation	Adjuvant dabrafenib + trametinib increased 5-year RFS rates (52% vs. 36%) and 5-year survival rates (52% vs. 36%)	Grade 3/4 adverse event rate: 36% vs. 10% in placebo	TBD	Adjuvant dabrafenib + trametinib approved for resected BRAF V600E/K mu stage III melanoma in 2018
COLUMBUS (Dummer et al., 2018) [40]	Binimetinib + encorafenib (*n* = 192) vs. encorafenib monotherapy (*n* = 194) vs. vemurafenib monotherapy (*n* = 191)	V600E/K-mutant, unresectable or metastatic melanoma	Encorafenib + binimetinib increased median PFS (14.9 mo vs. 9.6 mo with encorafenib only) and ORR (63% vs. 51%). At 5-year follow-up, combination therapy also demonstrated increased OS (33.6 mo vs. 23.5 mo with encorafenib only vs. 16.9 mo with vemurafenib only).	Encorafenib + binimetinib had lower rates of grade 3–4 adverse events (58%) than encorafenib alone (66%) or vemurafenib alone (63%)	Yes	Encorafenib + binimetinib approved for V600E/K mutant, unresectable or mM in 2018
EORTC1325/ KEYNOTE-054 double-blind phase III RCT (Eggermont et al., 2018 and Eggermont et al., 2021) [41,42]	Adjuvant pembrolizumab vs. placebo	Completely resected, stage III melanoma	Pembrolizumab improved 1-year RFS (75.4% vs. 61.0%) and reduced risk of recurrence or death by 43%	Rates of grade 3/4 adverse events were higher with pembrolizumab (14.7% vs. 3.4%)	Improved RFS; OS not yet determined	Pembrolizumab approved for adjuvant treatment for high-risk, stage III melanoma in 2019
IMspire150 double-blind phase III RCT (Gutzmer et al., 2020) [43]	Cobimetinib + vemurafenib+a tezolizumab (*n* = 256) vs. cobimetinib + vemurafenib+ placebo (*n* = 258)	Treatment-naïve, BRAF V600 mutant advanced or mM	Triple therapy improved PFS (15.1 vs. 10.6 mo, *p* = 0.025) and response durability (21.0 mo vs. 12.6 mo) without differences in ORR (66.3% vs. 65.0%)	Rates of grade 3/4 adverse events were similar (79% vs. 73%)	Not yet determined	Atezolizumab + vemurafenib + cobimetinib triple therapy approved for BRAF V600 mu unresectable or mM in 2020

FDA, (United States) Food and Drug Administration; RCT, randomized controlled trial; ECOG, Eastern Cooperative Oncology Group; IFN, interferon; RFS, relapse-free survival; OS, overall survival; RR, response rate; ORR, overall response rate; HD IL-2, high-dose interleukin-2; CRR, complete response rate; PRR, partial response rate; PFS, progression-free survival; PEG-IFN, pegylated interferon; mM, metastatic melanoma; SCC, squamous cell carcinoma; BRAF wt, wild-type; BRAF mu, mutant; Ipi, ipilimumab; Nivo, nivolumab; T-VEC, talimogene laherparepvec; DRR, durable response rate.

### 2.3. High-Dose Interleukin-2

Interleukin-2 (IL-2) is a T-cell growth factor that leads to cytokine production and preferential expansion of CD8+ T cells, NK cells, and Tregs. In 1998, HD intravenous (IV) administration of IL-2 became the first FDA-approved immunotherapy for the treatment of metastatic melanoma (mM) [25]. Durable tumor responses have been well documented in a subset of mM patients, with 5–10% of patients achieving complete response, and even more achieving increased disease stability [25,44,45]. A recent meta-analysis of IL-2-responsive mM patients who exclusively received HD IL-2 for systemic therapy confirmed prolonged clinical and survival benefits [46]. As with IFN-α therapy, the use of HD IL-2 treatment is limited by the relatively high incidence of grade 3 and 4 toxicities, which requires the drug to be administered in an intensive inpatient setting [47]. The efficacy of treatment is further limited by the drug’s activation of anti-inflammatory Tregs, which limit CD8+ activation and effector functions. Drugs targeting specific subunits of the IL-2 receptor, such as the recombinant IL-2 receptor βγ-biased agonist NKTR-214 (Bempegaldesleukin), have shown promise in targeted expansion of antitumor T and NK cells, with limited expansion of Tregs and dramatically reduced toxicity [48,49,50,51,52,53].

While rarely used as a single or first-line agent today, HD IL-2 remains a second- or third-line option that provides a possible survival benefit to patients who have failed treatment with first-line agents [54]. Many trials combining HD or low-dose IL-2 therapy with additional therapies are ongoing.

## 3. Targeted Therapies

### 3.1. Tyrosine Kinase Inhibitors

The discovery that B-Raf proto-oncogene (BRAF) mutations constitute the most prevalent (~50%) oncogenic drivers of melanoma revolutionized mM treatment [55]. The BRAF gene encodes a serine/threonine protein kinase that, when activated, gives rise to a cascade of phosphorylation events within the mitogen-activated protein kinase (MAPK) signaling pathway. After phosphorylation by mitogen-activated protein kinase kinase (MEK) 1/2, ERK 1/2 translocates to the nucleus and promotes cell growth, proliferation, and survival by modifying transcription factors and gene expression [56,57]. Over 97% of BRAF mutations occur in the Val600 codon of its kinase domain, with 90% involving a substitution of a valine for glutamic acid (BRAF^V600E^) [58]. This mutation locks BRAF in a constitutively active configuration independent of ligand-dependent receptor tyrosine kinase (RTK) activation. The result is aberrant signaling through the MAPK/ERK pathway. The second most common BRAF mutation involves a lysine substitution at the same position (BRAF^V600K^) and also results in constitutive activation [59]. The realization that a single pathway mutation may drive a significant portion of melanoma oncogenesis informed the efforts to design specific small-molecule inhibitors [55].

In 2011, almost a decade after the seminal BRAF mutation discovery, vemurafenib became the first selective BRAF inhibitor to be approved by the FDA as metastatic melanoma monotherapy. In a randomized phase III trial (BRIM-3), the selective oral BRAF^V600E^-mutant inhibitor demonstrated significantly improved PFS (5.3 months vs. 1.6 months), OS (84% vs. 64%), and ORR (48% vs. 5%) over dacarbazine chemotherapy [60]. A second BRAF^V600E^ kinase inhibitor, dabrafenib, was approved in 2013 after demonstrating similar improvements in PFS over dacarbazine (5.1 months vs. 2.7 months) [28].

### 3.2. MEK Inhibitors

While BRAF is the most common oncogenic mutation driving aberrant MAPK/ERK signaling, many other mutations exist, such as NRAS and NF1 [61]. Inhibitors targeting downstream substrates within the pathway, such as MEK, were developed to block a wider array of upstream oncogenic mutations. In 2013, the first MEK1/2 inhibitor, trametinib, was approved for BRAF-mutated metastatic melanoma after demonstrating improved PFS and OS compared to dacarbazine [27].

While a BRAF and MEK inhibitor monotherapy mark a significant improvement from pre-2010 mM treatment, efficacy is limited by the relatively rapid development of treatment resistance, usually within 7 months of treatment initiation [62]. Resistance can be driven by many mechanisms, including the acquisition of new mutations that recover MAPK/Erk signaling, activating mutations in the progrowth PI3K/Akt pathway, gene copy-number variation, altered gene-expression levels, metabolic reprogramming, and modulation of downstream signaling [63,64]. BRAF/MEK inhibitor monotherapies also paradoxically increase the risk of aberrant MAPK pathway signaling in nonmelanoma cells, increasing the rates of RAS-mutant tumor reactivation and secondary cancer development (e.g., cutaneous squamous-cell carcinoma) [62,65].

### 3.3. Combination BRAF and MEK Inhibitor Therapy

BRAF and MEK inhibitor combination therapy was developed to improve the efficacy of these medications and delay the onset of resistance. The combination of trametinib and dabrafenib was approved after demonstrating a 25% reduced risk of progression (i.e., reduced risk of resistance development) and improved ORR (67% vs. 51%; *p* = 0.002) compared to dabrafenib monotherapy in 2014 [62] and vemurafenib monotherapy in 2015 (COMBI-v trial) [29]. Adjuvant use of the trametinib and dabrafenib combination also demonstrated significant and durable improvements in RFS and distant metastasis-free survival compared to surgery alone in previously resected stage III BRAFV600E or K-mutant melanoma without increased toxicity [38,39].

Combination therapy with vemurafenib and cobimetinib, another MEK inhibitor, also increased PFS and ORR with a nonsignificant increase in toxicity compared to dabrafenib alone (coBRIM trial) [36]. Combination therapy also reduced the risk of therapy-induced secondary malignancies without increasing the rates of other severe toxicities [36,62]. After 2014, BRAF–MEK inhibitor combinations were considered the standard of care for BRAF-mutated metastatic melanoma, extending median PFS and OS to approximately 12 and 24 months, respectively [29,36,62].

Encorafenib is the newest FDA-approved BRAF inhibitor. It is an ATP-competitive BRAF inhibitor with a longer dissociation half-life and a wider range of selectivity for different BRAF mutations. In 2018, a trial of BRAF^V600^ -positive mM patients demonstrated that combination therapy of encorafenib with binimetinib, a MEK inhibitor, further increased PFS to 15 months and OS to 33.6 months compared to encorafenib monotherapy (PFS = 9.6 months; OS = 23 months) and vemurafenib monotherapy (PFS = 7 months; OS = 17 months) [40,66].

While trials testing different combination therapies are still ongoing, no one combination of BRAF and MEK inhibitors is clearly superior to the others for BRAF-mutant metastatic melanoma [67]. As such, three BRAF/MEK combination therapy options are recommended by the most recent (2020) ASCO guidelines on systemic therapy for BRAF-mutant metastatic or unresectable melanoma: dabrafenib/trametinib [38,39], encorafenib/binimetinib, or vemurafenib/cobimetinib. The guidelines also give equal recommendation for three other immunotherapies—ipilimumab plus nivolumab, nivolumab alone, or pembrolizumab—which can be used in both BRAF wild-type and BRAF-mutant disease [68]. Choosing between recommended options involves consideration of patient comorbidities, anticipated toxicities, previous treatments, and preferences for treatment administration. In the future, predictive biomarker analysis for each patient may also aid in this choice. However, guidelines may change before that point due to recent evidence from IMspire 150, the first trial to combine ICIs and a BRAF–MEK inhibitor combination therapy. In this phase III trial, the addition of atezolizumab, an anti-PD-L1 antibody, to vemurafenib–cobimetinib combination therapy significantly increased PFS over 19-month follow-up in patients with BRAF^V600^-mutated mM (15.1 vs. 10.6 months; [HR] 0.78; 95% CI 0.63–0.97; *p* = 0.025) without increasing toxicity [43]. This study led to the approval of this combination therapy for BRAF-mutated unresectable or metastatic melanoma. These data further support the rationale of using a multimeric therapeutic approach to combine drugs with high response rate (i.e., BRAF and MEK inhibitors) with drugs with a high durability (i.e., checkpoint inhibitors). Future treatment recommendations may change once survival data become available from ongoing trials.

### 3.4. Other Targeted Therapies

Mutations in c-KIT, a proto-oncogene RTK, are drivers of mucosal and acral melanomas [61]. Trials with the tyrosine kinase inhibitor imatinib in KIT-amplified melanomas demonstrated a relatively high RR (53%), but a limited PFS (3.9 months) and no tumor regression [69]. Imatinib efficacy is likely limited by the presence of other driver mutations [61], suggesting that combination therapy may improve results. This has led to the initiation of a trial combining nilotinib, a BCR-ABL tyrosine kinase inhibitor designed to overcome imatinib resistance, with dabrafenib and trametinib for BRAF-mutant and BRAFi-resistant mM (NCT04903119).

Other small-molecule inhibitors in clinical trials include: IN10018, a focal adhesive kinase 1 (FAK1) inhibitor designed to suppress the AMBRA-1 (autophage/beclin regulator 1) pathway key to melanoma invasive capacity [70,71,72] (NCT04109456); SX-682, an oral CXCR1/2 chemokine receptor inhibitor that may prevent myeloid-derived suppressor cells from mediating immunosuppression within the TME (NCT03161431); tazemetostat, an EZH2 histone methylase inhibitor, which may prevent the silencing of tumor suppression genes, induce direct tumor cytotoxicity, and increase ICI efficacy [73] (NCT04557956); APG-115, an MDM2 antagonist with p53-activating abilities that may augment PD-1 inhibitor therapy by modulating the TME macrophage population, stimulating T cells, and increasing tumor cell PD-1 expression (NCT03611868) [74,75]; and palbociclib, a CDK4/6 inhibitor that may slow the development of BRAF/MEKi resistance or provide alternative therapy for BRAF/MEKi-resistant disease [76] (NCT04720768).

Bispecific antibodies may also change the landscape of small-molecule inhibitors by offering higher-specificity targeting and a mechanism to bypass some tumor-resistance mechanisms. A new bispecific antibody targeting T-cell CD3 and TYRP1, a protein overexpressed in over 50% of mM tumor cells, allows directs tumor–T-cell engagement without requiring MHC presentation or an APC [77]. AT1413 bTCE, another antibody that binds bivalently to CD43s and CD3ε, has demonstrated significantly reduced tumor outgrowth, increased T-cell numbers, and increased T-cell activation in preclinical models [78].

## 4. Immune-Checkpoint Inhibitors

### 4.1. Cytotoxic T Lymphocyte-Associated Antigen 4 (CTLA4) Inhibitors

CTLA4 is an immune-inhibitory molecule expressed on the surface of activated T cells. Together with its immune-activating counterpart, CD28, CTLA4 creates a critical immune checkpoint that must be overcome to achieve a durable immune response [79]. CTLA4 is naturally upregulated in situations of chronic T-cell stimulation to prevent uncontrolled immune reactions and inappropriate development of autoimmunity. In the TME, however, this system backfires: chronic presentation of tumor antigens to T cells inhibits the immune system from mounting an antitumor immune response and contributes to the immune evasion that allows continued tumor growth [80,81,82,83].

Ipilimumab was approved as the first immune-checkpoint inhibitor (ICI) in 2011, the same year that vemurafenib was approved to block BRAF-mediated growth signaling. Ipilimumab is an anti-CTL4 human IgG antibody. By preventing the interaction of CTLA4 and its ligands, the drug allows T cells to bypass the inhibitory immune checkpoint and mount a response against tumor antigens. Phase III trials of previously treated mM patients demonstrated improved OS compared to gp100, a melanoma antigen immunostimulant with limited antitumor effects (10.1 vs. 6.4 months; *p* = 0.0026) [26]. A metanalysis of pooled data from nearly 2000 mM patients treated with ipilimumab (both pretreated and treatment-naïve) reported an increase in the 3-year OS rate to 22% (95% CI (20, 24%)), a dramatic increase from ~5% achieved by previous standard-of-care therapies [84]. Perhaps even more importantly, the OS survival curve plateaued after 3 years, maintaining the ~20% OS rate for the entirety of the 10+ year follow-up [84]. Thus, ipilimumab became both the first therapy to provide an OS benefit in advanced melanoma, and the first to demonstrate that long-term durable mM disease control is possible with systemic therapy [85].

While responses to ipilimumab are durable, the response rates are low, ranging from 5% to 10%. Clinical trials have provided little insight into possible biomarkers of response. Attempts to improve response rates by adding ipilimumab to dacarbazine therapy were somewhat successful (15% vs. 10%) and demonstrated a survival benefit over dacarbazine alone (OS 11.2 vs. 9.1 months; *p* < 0.001). However, these benefits came at the cost of high toxicity (rate of grade 3/4 AEs: 56.3% vs. 27.5%; *p* < 0.001) [86]. Even as a monotherapy, ipilimumab is relatively toxic, with immune-related toxicities occurring in 60–80% of patients, 10–26% of which are grade 3/4 reactions [87]. Perhaps unsurprisingly, severe AEs, which are often immune-related AEs (irAE), were found to be associated with improved ORRs [88].

Ipilimumab is still the only approved CTLA4 inhibitor for mM after the initially promising drug tremelimumab failed to demonstrate improved OS (*p* = 0.127) or RR over standard-of-care chemotherapy, despite a longer average response duration (35.8 vs. 13.7 mo; *p* = 0.0011) [89]. Today, ipilimumab monotherapy is not a first-line therapy by ASCO guidelines [68].

### 4.2. Programmed Cell Death Protein 1 (PD-1) and PD-1 Ligand (PD-L1) Inhibitors

Like CTLA4, PD-1 is an inhibitory immune checkpoint receptor expressed by activated T cells. When PD-1 binds its receptors, PD-L1 and PD-L2, signaling through the SHP1/2 pathway downregulates the transcription factors necessary for T-cell effector functions, growth, and survival [90]. In healthy tissues, PD-L1 is broadly expressed and upregulated in response to proinflammatory cytokines [91]. Melanoma tumor and TME cells upregulate PD-L1 in response to tumor-infiltrating lymphocytes (TIL), suggesting that PD-L1 expression is used as a mechanism of immune evasion by the cancerous cells [92,93,94].

In 2014, two anti-PD-1 monoclonal antibodies, pembrolizumab and nivolumab, were approved for treatment-resistant mM after demonstrating superiority over ipilimumab. Early trials of pembrolizumab monotherapy demonstrated improved 6- and 12-month PFS and RR (6-month PFS: pembrolizumab = 47% vs. ipilimumab = 26%; 12-month PFS: P = 74–68% vs. I = 58%; RR = P = 33%, I = 12%) [42,95]. Two- and five-year follow-up studies and real-world findings of pembrolizumab monotherapy confirmed its superior OS and durable antitumor immune activity for both treatment-naïve and pretreated mM patients [31,96,97,98,99]. Similarly, the CheckMate 067 trial first demonstrated that nivolumab monotherapy confers a significantly greater PFS compared to ipilimumab in treatment-naïve mM patients (nivolumab: 6.9 months 95% (4.3, 9.5); ipilimumab: 2.9 months 95% (2.8, 3.4) [34]. Follow-up data from 2019 then demonstrated superior 5-year OS rates (nivolumab = 44%, ipilimumab = 26%) [33]. Nivolumab has proven to be effective in a range of melanoma tumor subtypes, including both treatment-naïve and pretreated tumors with either WT or mutant BRAF status [30,100].

The two PD-1 inhibitors differ by epitope binding location and target affinity strength, but are equally effective as monotherapies by OS (pembrolizumab = 22.6 mo, nivolumab = 23.9 mo; *p* = 0.91) and time to next-line therapy or death (pembrolizumab = 15.7 mo, nivolumab = 10.8 mo; *p* = 0.16) [101,102]. Both are also relatively well-tolerated, with lower rates of grade 3/4 toxicity (14% with pembrolizumab and 4% with nivolumab) than chemotherapy, ipilimumab, and most targeted therapies [101,102]. AEs during nivolumab therapy were associated with improved ORRs [102].

Both PD-1 inhibitors are also effective in the adjuvant setting. A five-year study on adjuvant pembrolizumab demonstrated a significantly increased RFS, a decreased risk of distant metastasis or death (HR 0.60 95% (0.49, 0.73)), and a sustained treatment effect compared to placebo [32,41]. Interestingly, adjuvant pembrolizumab also proved efficacious in patients with PD-L1-negative and undetermined tumors [41,42]. In pretreated stage IV melanoma patients with no evidence of residual disease, adjuvant nivolumab alone or in combination with ipilimumab proved similarly effective in increasing RFS compared to placebo [103]. These drugs are the current first-line adjuvant therapy for resected WT melanoma. Patients with resected BRAF-mutant melanomas may choose between pembrolizumab, nivolumab, or dabrafenib–trametinib combination therapy as first-line adjuvant therapy [68].

Optimal utilization of ICIs is hindered by several major challenges, including resistance and poor predictability of patient response. Approximately 30% of melanoma patients have innate resistance to PD-1 inhibitors, and 25% of responders acquire resistance during treatment [104,105,106]. CTLA4 inhibitors face a similar challenge [26]. Mechanisms of resistance likely include specific tumor-cell genetics (loss-of-function mutations in *JAK1/2* [107]), and differing expression levels of tumor-cell surface proteins (e.g., MHC I expression [108], alternate ICIs [109], and epigenetic T-cell changes limiting effector function and memory [110]). Efforts to increase durability by combining ICIs with auxiliary agents such as PEG-IFN [111] or hydroxychloroquine [112] have had mixed results, with none providing a clear clinical benefit. Unfortunately, increased toxicity often outweighs any benefit to durability or RR that auxiliary agents provide.

A few studies have identified markers associated with more successful clinical outcomes. For example, independent biomarkers associated with favorable OS of mM patients treated with pembrolizumab include a high relative eosinophil count (>1.5%), a high relative lymphocyte count (>17.5%), and absence of non-soft tissue or lung metastasis. Patients meeting none of these criteria have a poor prognosis with pembrolizumab [113]. Others have identified that the occurrence of immune-mediated AEs may be associated with better ORR, OS, and PFS with nivolumab and ipilimumab monotherapies, but not with pembrolizumab [114]. Another—albeit much smaller (*n* = 40)—study also found that PD-L1 expression on circulating tumor cells may also be a predictive biomarker for PD-1 inhibitor response, suggesting that liquid biopsy may provide clinically relevant information during treatment selection [115]. However, subgroup analyses have demonstrated the PD-1 blockade still provides clinical benefit in PD-1 negative tumors [34]. Conflicting evidence on the subject makes using tumor PD-L1 expression as a predictive marker for PD-L1 inhibitor response or overall prognosis for mM controversial [116,117].

### 4.3. Combination ICI Therapy

Combining CTLA4 and PD-1 blockade is more effective than either class in monotherapy [118,119], yet carries a significantly higher risk of severe toxicity. As monotherapies, nivolumab and ipilimumab have grade 3/4 AE rates of 16–27% and 27%, respectively. When used together, this rate increases to 55–71% [34,103]. Reducing toxicity while maintaining the clinical benefit of combination therapy may be possible with alternative dosing strategies. Regimens of standard-dose pembrolizumab (200 mg) with either 150 mg or 50 mg reduced-dose ipilimumab showed a meaningful reduction in toxicity (grade 3–5 toxicity rate <26%) without a significant reduction in ORR (PEM200+IPI50: ORR, 55%, and CR, 16%; PEM200 + IPI100: ORR, 61%, and CR, 25%). In fact, 12-month PFS and OS rates were actually higher with these regimens (12-month PFS: 65% for PEM200+IPI50; 82% for PEM200+IPI100; OS: >90% for both) compared to standard dosage and previous alternative dosages (12-month PFS: 46–53% with standard dosing, 47% and 68% with alternative dosing; 12-month OS: 73–89%) [120,121,122,123,124]. Larger trials are still necessary.

### 4.4. Novel Immune Checkpoint Inhibitors

The second generation of PD1 and CTLA4 ICIs are emerging. HX008, a new anti-PD1 antibody, demonstrated equal efficacy and reduced toxicity compared to nivolumab in pre-clinical trials [125], and has been well tolerated in early clinical trials [126]. HX008 in combination with the novel PD-L1 monoclonal antibody LP002 is entering clinical trials as a potential synergistic regimen for overcoming PD1 resistance in previously treated mM patients (NCT04756934). New agents may also soon emerge for CTLA4 blockade. Recent work suggested that separate mechanisms mediate the clinical efficacy and the toxicity of CTLA4 antibodies. This has allowed for the creation of an anti-CTLA4 antibody (ONC-392) that is highly selective for the immunotherapeutic mechanism. In preclinical data, this antibody maintains selective depletion of Tregs while preserving CTLA4 recycling, the mechanism responsible for irAEs [127]. Initial phase I trials were promising and demonstrated no irAEs [128]. Phase I/II trials are currently recruiting (NCT04140526) [129].

Lymphocyte activation gene 3 (LAG3) is another T-cell inhibitory checkpoint receptor, the upregulation of which may be a resistance mechanism to PD1 inhibition therapy [130]. An ongoing phase III study (RELATIVITY-047; NCT03743766) of the new anti-LAG3 antibody relatimab has demonstrated promising initial results. Patients receiving combination relatimab and nivolumab achieve significantly higher rates of 12-month PFS (47 vs. 36%; *p* = 0.0055) than those receiving nivolumab alone [131]. Rates of severe toxicity were higher in the combination group (19% vs. 9.7%), but were lower than the toxicity rates previously reported with combination CTLA4/PD-1 inhibition [34,103,131]. Further investigation is needed to determine the effects on response rates and overall survival.

Another promising ICI target is TIM-3 (T-cell immunoglobulin and mucin domain 3). TIM-3 blockade restored antitumor functions in ex vivo studies of previously exhausted NK and effector T cells [132] and enhanced cancer-vaccine-induced antitumor responses in murine melanoma models [133]. A bispecific anti-PD-1 and TIM-3 antibody (RO7121661/RG7769) demonstrated superior antitumor TIL activity, IFN-γ secretion, and tumor growth control compared to the monospecific PD-1 antibody in mouse models [134]. The agent has recently entered phase I human trials (NCT03708328).

## 5. Oncolytic Virus Therapy

Cancer cells achieve a neoplastic phenotype by genetic and epigenetic mutations. These mutations, however, impair signaling pathways (*RAS, WNT, PTEN, RB1, TP53*) that are essential for the intracellular antiviral response [135]. Recent advances in genetic modification, such as CRISPR, have allowed researchers to create antineoplastic viruses that exploit the vulnerability of mutated cells to viral infection while sparing healthy cells [136].

Talimogene laherparepvec (T-VEC), an oncolytic human herpes simplex virus 1 (HSV-1), is the first and only oncolytic virus (OV) approved for metastatic and unresectable melanoma. When T-VEC is injected directly into the tumor site, it promotes the secretion of granulocyte-macrophage colony-stimulating factor (GM-CSF) to activate DCs and increase tumor antigen presentation to T cells. In the phase 3 OPTiM trial, 64% of directly injected and 34% of uninjected nonvisceral lesions decreased in size by >50%. Complete resolution of lesions occurred in 47% of injected lesions, 22% of noninjected nonvisceral lesions, and 9% of noninjected visceral lesions. When compared to recombinant GM-CSF administration, T-VEC demonstrated a higher durable RR (16% vs. 2.1%, *p* = 0.001), ORR (26% vs. 5.7%), and OS (23.3 months, *p* = 0.051). Severe toxicity rates were only 2% [35]. Laboratory evidence showed that T-VEC has increased efficacy in melanomas with INFγ–JAK–STAT pathway mutations [137]. Since dysregulation of INFγ is a common mechanism of resistance to ICI therapy, ongoing trials are investigating T-VEC as a salvage or combination therapy (NCT04330430, NCT04068181).

Systemic administration of OV therapy is also being explored. However, maintaining viral titers capable of generating an antitumor response after systemic administration has proved challenging to systemic OV monotherapy [138,139]. Trials are also investigating their role as sensitizing agents or within combination immunotherapies. Systemic OVs may still have a role as priming agents or within combination therapy (NCT04152863).

Promising new antimelanoma oncolytic viruses are in various stages of development. A targeted, inflammation-inducing vesicular stomatitis virus (VSV-GP) has shown promise in animal models [140]. A CD40 ligand and IFN-beta dual-transgene-armed adenovirus (MEM-288) and a tumor-selective TNF-alpha and IL-2-expressing adenovirus (TILT-123) are in stage I clinical trials (NCT05076760, NCT04217473) [141,142]. A promising intralesional Coxsackievirus A21 V937 may soon move to phase III trials [143]. Other OVs, such as ONCOS-102, have limited efficacy as monotherapy, but may provide clinical benefit by enhancing the effects of coadministered immunotherapies [144].

Personalized OVs, targeting each patient’s unique constellation of neoantigens, may be the future of OV therapy. A live-attenuated *Listeria monocytogenes* (ADXS-NEO) that secretes a personalized viral-neoantigen fusion has already demonstrated effective activation of antitumor T cells and inhibition of TME myeloid-derived suppressor cells and Tregs in preclinical studies [145,146]. Human trials have since been approved for initiation (NCT03265080).

## 6. Melanoma Vaccines

The five major categories of melanoma vaccines currently in development include: (1) melanoma-cell-targeted vaccines, (2) dendritic cell (DC) vaccines, (3) peptide-based vaccines, (4) vector-based vaccines, and (5) mRNA or DNA vaccines. Unlike preventative immunizations, cancer vaccines are therapeutic, activating the patient’s immune system to incite an antitumor response against a known cancer or to prevent disease recurrence in the adjuvant setting.

Whole-cell vaccines use modified melanoma cells to activate a complex immune response to the cell’s melanoma antigens. In theory, using a whole-cell approach exposes the immune system to many potential antigens, circumventing the need to identify the most immunogenic antigens for each tumor [147]. Most of the cells used in these vaccines are also genetically modified to express immunostimulatory molecules because irradiated tumor cells are not typically immunogenic on their own [147]. In the past, most whole-cell vaccines were made using allogenic cells. Once technical challenges are addressed, we may be able to use autologous tumor cells to create individuated vaccines for each patient. While several melanoma cell vaccines have shown promise in the laboratory, clinical efficacy has so far been limited [148].

DC vaccines are used to directly inject activated or modified DCs into the tumor site to increase antitumor T-cell activation. While these vaccines have shown some immunogenicity in murine models, efficacy has not yet been demonstrated in human trials, likely due to the immunosuppressive nature of the tumor microenvironment [149,150].

Peptide vaccines supply tumor-specific or tumor-associated antigen (i.e., gp100, MART-1/MelanA, tyrosinase) fragments that can be presented by professional APCs to induce effector T-cell activation. Despite promising laboratory data, short-peptide vaccines have shown limited clinical efficacy, even with adjuvant immunostimulatory agents. Long-peptide vaccines with adjuvant immunostimulatory agents are currently under investigation [151]. In 2018, a phase I trial of eight high-risk melanoma patients demonstrated the safety and immunogenicity of a personal neoantigen long-peptide vaccine administered in the adjuvant setting [152]. Four years later, and despite several relapses, all maintained neoantigen-specific T-cell responses and showed evidence of tumor infiltration by neoantigen-specific, cytotoxic T-cell clones [153]. A study evaluating the feasibility of a similar personal neoantigen vaccine in combination with anti-PD1 therapy as a first- or second-line therapy is ongoing (NCT03715985).

Vector vaccines use recombinant viral vectors to deliver tumor antigen transgenes directly to APCs. Within the APCs, the transgenes are expressed to produce high concentrations of tumor antigens that can be presented on both MHCI and MHCII for enhanced T-cell activation. The simultaneous expression of viral proteins by the delivered vectors boosts the immunogenicity of the vaccine [154].

Vaccines that use mRNA have garnered significant excitement as therapeutics after the advancements and efficacy demonstrated in the COVID-19 pandemic response. While still in the early stages of research, mRNA vaccines may have the potential to induce the targeted expression of nearly any protein. Using an mRNA approach avoids safety concerns associated with DNA and viral vector vaccines. Therapeutic mechanisms under investigation include enhancing the expression of tumor-specific antigens in DCs, mRNA-mediated delivery of specific antitumor or anti-ICI antibodies, and programming cancer cells to express suicidal intracellular proteins [155,156,157]. Challenges include mRNA instability, low in vivo translation rates, and autoimmune reactions [157].

## 7. Photodynamic Therapy

Photodynamic therapy (PDT) involves the administration and subsequent activation of tumor-cell-targeted photosensitizers (PS) (i.e., porphyrin, phthalocyanines, chlorins, porphycenes). When these photosensitizers are activated by light of a specific wavelength, cytotoxic and immunostimulatory reactive oxygen species (ROS) are generated in tumor tissues and tumor-associated blood vessels [158]. Since ROS are only produced in the presence of both the photosensitizer and the light source, this therapy has the potential to induce localized tumor destruction with limited off-target effects. Preclinical studies of PDT have demonstrated enhanced antitumor immune responses in lung and nonmelanoma skin cancer [159]. Early human clinical trials in head and neck cancers and basal cell carcinomas suggest a therapeutic benefit [160,161]. In melanoma mouse models, there is some evidence of survival benefit after PDT, as well as synergism with PD1 inhibitor therapy, suggesting a potential for use in the adjuvant setting in humans [129,158,162,163].

Despite promising preclinical results, PDT faces significant limitations for use in mM. Melanoma cells are innately resistant to PDT due to pigmentation and enhanced ROS tolerance [164]. Furthermore, light sources reach deeper tumor cells at significantly lower levels, if at all. Photosensitizing agents have also yet to be consistently optimized for efficient delivery and specificity in order to avoid off-target effects. Studies are ongoing to address these issues, with a particular interest in nanoparticle delivery systems and nanoemulsions [165]. One such study found that using acai oil in a nanoemulsion (NanoA) as a photosensitizer induced apoptosis in 85% of melanoma tumor cells, with an 82% reduction in tumor volume while maintaining high viability in healthy cells in in vitro and in vivo murine models [166].

## 8. Toll-like Receptor Agonists

Toll-like receptor (TLR) activation and the resulting proinflammatory cytokine release are critical steps in the induction of both the innate and adaptive immune response [167]. As poor immunogenicity continues to limit the efficacy of melanoma immunotherapies in some patients, TLRs are a logical ancillary agent that provides proinflammatory modulation of the tumor microenvironment. Small combination studies have so far demonstrated that TLR agonists induce palliative local disease control for inoperable mM when combined with intralesional IL-2 [168], increased local and systemic antimelanoma immunity with monobenzone [169], and increased total and vaccine-specific CD8+ T cells with a multipeptide melanoma vaccine [170]. Another clinical trial demonstrated increased plasmacytic DC activation and increased tumor regression when the TLR7/8 agonist resiquimod was coadministered with a peptide vaccination [171]. Clinical trials further investigating the immunostimulatory properties of TLR agonists in the context of melanoma immunotherapy are ongoing (NCT03276832, NCT04072900, NCT02394132, NCT03684785). Laboratory evidence also suggests that TLR agonists may boost the efficacy of targeted small-molecule inhibitors [172]. In mouse models, coadministration with targeted BRAF inhibitors preserved immunogenicity and delayed treatment resistance [173].

RIG1-like (retinoic acid-inducible gene-1) receptor (RLR) agonists and melanoma differentiation-associated antigen 5 (MDA-5) agonists are two other potential immunostimulatory targets for use in combination therapy. In mouse models, these proapoptotic molecules have proven capable of overcoming the characteristic resistance of melanoma cells to apoptosis [174].

## 9. Adoptive Cell Transfer with Tumor-Infiltrating Lymphocytes

In melanoma, the presence of tumor-infiltrating lymphocytes (TILs) is associated with a more favorable OS, RFS, and DSS/MSS [175]. Prognosis improves as TIL cell count and the physical area of infiltration increases, likely because these metrics reflect an effective antitumor immune response [175]. As discussed above, high-dose IL-2 therapy is used to expand melanoma TILs in vivo. Adoptive cell transfer (ACT) of TILs is the process of expanding autologous lymphocytes in vitro, usually aided by IL-2, IL-7, IL-15, and/or IL-21, followed by reinfusion to the patient [176]. This strategy circumvents many limitations of other immunotherapies. For example, in vitro TIL culture allows for the selective expansion of lymphocytes with the strongest effector function and the highest tumor–antigen affinity. Using autologous cells from resected tumor specimens avoids issues of rejection and allows each treatment to be uniquely targeted to the patient’s specific tumor antigens [177]. Since expansion and activation occur without the suppressive effects of the TME, higher numbers of activated lymphocytes (>10^11^ TILs) can be achieved. This also allows for pretreatment manipulation of the patient’s immune system to optimize the efficacy of ACT or other planned immunotherapies without compromising the antitumor response. Greater response rates have been achieved when lymphodepletion proceeds and IV IL-2 follows ACT, both of which promote T-cell homeostatic cytokine production [178,179,180].

Since TIL–ACT regimens are not yet standardized, the degree of treatment efficacy reported in clinical trials has varied. Disease progression and overall survival after ACT–TIL are dependent on the expansion of neoepitope-specific CD8+ T cells [181]. ORRs typically range from 28% to 45% [176]. The highest ORRs achieved using TIL–ACT were 49%, 52%, and 72% using lymphodepleting chemotherapy alone, or combined with 2 or 12 Gy TBI, respectively, in heavily pretreated mM patients. Complete response was initially observed in 22% of patients [182]. Five-year follow-up found notable durability and suggested curative potential. Of the 22% CRs, all but one remained disease-free after 3 years, resulting in 100% 3-year and 95% 5-year survival rates [182]. It is especially exciting that these results occurred in challenging mM cases, in which patients had a median of 3 metastatic sites and had all failed first-line treatments, including 20% who had failed ICI therapy. However, it should be noted that TBI is no longer used in patient preparation for ACT [179]. In another longitudinal ACT study, 46 of 48 single-dose ACT complete responders maintained ongoing responses for at least 7.5 years and had a 10-year melanoma-specific survival of 96% [183].

Patients who receive TIL–ACT after failing ICI treatment have lower ORRs (56% vs. 24%) and OS (28.5 vs. 11.6 months) than ICI-naïve patients [183]. The same is true for patients with BRAF V600E/K mutations who failed prior targeted therapy (ORR: 21% vs. 60% if naïve; OS 9.3 vs. 50.7 months) [183]. This is likely because the poor immunogenicity and complex resistance mechanisms that allow tumors to evade ICIs also limit the efficacy of TIL–ACT [176]. However, an ongoing study of TIL–ACT in treatment-resistant mM has demonstrated an 80% disease control rate. Considering the higher toxicity rates and similar response rates of other second-line treatments, such as nivolumab or ipilimumab, TIL–ACT may be the best option for some patients resistant to alternative treatments [184,185].

Limitations to ACT–TIL are similar to those of other immunotherapies. As discussed above, resistance remains a central issue. Similarly, target identification, predictability of immunogenicity, and antitumor specificity (sparing healthy tissues) are essential for ACT–TIL success, but solutions remain in the early stages of development. Protocols for TIL expansion, antigen identification, pretreatment immunodepletion, and postinfusion TIL maintenance (e.g., IL-2 dosing) must be optimized for time, cost, efficacy, and safety in order to make this therapy feasible on a larger scale. A novel study that aims to improve ACT–TIL durability is investigating whether ex vivo treatment of TIL with a novel Myc–TAT (trans-activator transcription) fusion protein can prolong survival and promote proliferation of these cells after patient reinfusion (NCT03385486), potentially limiting the need for IL-2 infusions, which must be administered at high acuity centers.

## 10. ACT with T-CARs

Another approach to ACT utilizes autologous T-cells modified ex vivo with cell-surface chimeric antigen receptors (CAR-T cells). The extracellular component of the CAR is a variable region of a synthetic antibody. It is attached to a T-cell signaling moiety and costimulatory domains, which allows MHC-independent T-cell activation [186]. CAR-T cells can thus target tumors cells that have downregulated MHCs as an immune-escape mechanism [187].

Success with CAR-T ACT for the treatment of hematologic malignancies sparked the investigation into the therapy for solid malignancies. However, success in mM clinical trials has been limited. An early trial with vascular endothelial growth factor receptor-2 (VEGFR-2)-targeted CAR-Ts was terminated due to limited objective response [188] (NCT01218867). A phase I trial of anti-GD2 CAR-T was completed in 2019, but has not reported any conclusive results (NCT02107963). High rates of toxicity and evidence of rapid TME resistance to the therapy were discouraging [189,190]. However, recent advances in avoiding tumor resistance and limiting toxicity have renewed interest in CAR-T technologies [188]. Phase I trials of CAR-T cells targeting IL13Rα2 (NCT04119024), CD20 (NCT03893019), and B7-H3 (NCT05190185) are currently recruiting. Preclinical studies have identified several promising potential future targets, including CD126 [191]; chondroitin sulfate proteoglycan 4 (CSPG4), also known as MCSP [192]; tandem CD70 and B7-H3 [193]; and α_v_β_3_ integrin [194].

## 11. Radiotherapy

For many years, radiation was considered an immunosuppressive therapy [195]. However, recent studies have demonstrated that radiotherapies induce complex immunologic effects. Locally, radiation therapies modulate the TME by increasing inflammatory cytokine production, APC and CD8+ T-cell activation, and sensitizing tumor-supporting stromal cells to T-cell-mediated destruction [196,197,198,199]. Radiation also induces direct cancer-cell DNA damage, MHC I expression, and Fas cell-death ligand expression by cancer cells, increasing both the recognition and susceptibility of cancer cells to effector T and NK cells [200,201,202,203,204,205]. Immunotherapies achieve higher response rates and efficacy in immune-activated TMEs [206]. Taken together, these effects contribute to enhanced innate and adaptive antitumor immunity, and suggest synergistic potential with immunotherapies. The combination may also enhance the effects of RT, specifically by increasing the incidence of the “abscopal effect,” a rare immune-mediated response to radiation that results in tumor regression at nonradiated metastatic sites [207,208].

However, before the advent of immune checkpoint inhibitors, neither local nor whole-body radiotherapy provided a consistent additional benefit. Combining IFN-α2b with fractionated radiotherapy has been studied many times in mM patients, but has been complicated by high rates of toxicity without clear long-term benefits [209,210,211]. Despite the suggestion by a phase I trial that stereotactic body radiation therapy (SBRT) and subsequent HD IL-2 in patients with mM demonstrated improved response rates compared to historical HD IL-2 monotherapy data (71% vs. ~15%) [25,212], phase II trials have not yet demonstrated a significant benefit over IL-2 alone [213].

Combining targeted immunotherapies with RT, however, has demonstrated more promising results. Preclinical models strongly support the synergistic efficacy of RT, anti-CTLA4, and anti-PD-L1 antibody triple therapy [202,214,215,216]. The combination also seems safe, with multiple trials demonstrating that concurrent anti-PD-L1 or anti-CTLA-4 therapy with focal palliative RT, whole-body radiotherapy (WBRT), or stereotactic radiosurgery are well tolerated without significantly increased rates of grade 3/4 toxicities [217,218]. Efficacy, however, is controversial. Two recent multivariate analyses including 451 and 936 melanoma patients found improved survival in those receiving combination therapy compared to either RT or immunotherapy alone [219,220]. Another single study demonstrated that concurrent anti-PD-1 therapy and fractionated RT resulted in a significantly higher ORR (64.7%% vs. 33.3%, *p* = 0.02) without significant differences in 6-month disease-free survival and OS [221,222]. However, a high number of mM patients with brain metastases were included in all three of these studies. This may be a significant confounder in these studies, since combination therapy may be particularly effective in this historically difficult population to treat [223,224,225]. Further study is also needed to determine the best immunotherapeutic agents, order and time intervals at which to administer each treatment, radiation dosing and fractionation, and site of irradiation. Over 50 clinical trials are currently underway to evaluate RT and immunotherapy in various stages of mM treatment [226].

## 12. Future Outlook

Over the past 8 years, immunotherapy has revolutionized the treatment of mM, offering patients more treatment options with higher efficacy and less toxicity. Two-year overall survival rates have risen dramatically from ~10% to ~60% [33]. Further research into the identification of melanoma neoantigens and their immunogenic potentials is essential for the advancement of the field. The ability to create individualized therapies specific to each patient’s tumor and immune landscape has the potential to revolutionize melanoma therapy. However, significant advances in rapid tumor-cell sequencing and vaccine production must first be achieved. In the shorter term, combination therapy and melanoma vaccines show promise for improving the efficacy, response rates, and durability of current first-line immunotherapies.

### 12.1. Sequencing and Combining Therapies

Using combined therapies to treat mM may be the easiest way to achieve longer-lasting disease control, overcome innate resistance, evade adaptive resistance to immunotherapy, and optimize clinical response. There is significant interest in finding the best combinations of the two most effective approved therapy classes—targeted and ICI therapy (NCT02631447, NCT03235245, NCT02902029, NCT02224781). Such studies may also address the two major roadblocks in the deployment of these therapies: rapid resistance development and modest response rates. Other studies are investigating combinations of approved and experimental therapies, including combinations of immunotherapy plus KIT inhibitors [227,228,229], VEGF inhibitors (NCT02681549, NCT01950390, NCT00790010) PI3K–AKT–mTOR pathway inhibitors (NCT02646748, NCT03131908), cyclin-dependent kinase (CDK) inhibitors (NCT02791334) [230,231], FGFR1-3 inhibitors (NCT02159066), propranolol (NCT03384836), and even fecal microbiota transplant [232]. Interesting experimental therapeutics beginning to be explored in human trials include a small molecule that selectively kills cancer cells that express elevated levels of specific proteins (NCT04809805) [233] and a device that removes soluble tumor necrosis factor receptors (sTNFR) during apheresis to reduce tumor-induced immunosuppression (NCT04142931) [234].

Consideration of quality-of-life markers must also be included when determining optimal therapy regimens, as some highly effective immunotherapies carry high rates of toxicity that may become further amplified in certain contexts. For example, a 2019 trial demonstrated that while dabrafenib, trametinib, and pembrolizumab triple therapy may increase PFS compared to dabrafenib + trametinib doublet therapy, it also increased AEs from 26.7% to 58.3%, an unacceptable toxicity profile for many patients [235].

### 12.2. Neoadjuvant Therapy

Neoadjuvant therapy is typically used to reduce tumor burden and allow for a less-extensive surgeries. Before immunotherapy, neoadjuvant systemic therapy was not the standard of care for mM treatment, likely because the risks of delaying surgery outweighed the limited benefits these therapies could provide. However, preclinical data suggest that this may not be true for immunotherapy [236], especially for therapies targeting T-cell function and proliferation. Theoretically, initiating immunotherapy while the major tumor mass is still present may induce a stronger antitumor T-cell response. Indeed, a small feasibility study confirmed these results, demonstrating that patients receiving neoadjuvant and then adjuvant treatment had significantly more expansion of tumor-resident T-cell clones than patients who received the same treatment courses exclusively as an adjuvant [237]. Neoadjuvant immunotherapy also seemed to outperform adjuvant therapy in comparative studies with event-free-survival benefit [238]. However, the sample size was small, and the toxicity profile of the neoadjuvant arm was disappointing. Larger trials are currently underway to investigate neoadjuvant regimens that preserve efficacy while limiting toxicity (NCT02977052), with promising initial results [239].

Response data from these early studies also suggest that neoadjuvant therapy provides helpful markers for prognosis. A pathological complete response to neoadjuvant immunotherapy, for example, correlates with significantly improved RFS and 5-year OS [239,240,241]. The extent of neoadjuvant response observed can also inform a more effective choice for adjuvant therapy. Pathological specimens may also inform adjuvant therapy choice by providing insight into each patient’s specific tumor adaptations, mechanisms of resistance, and biomarkers, such as postsurgical tumor mutational burden and IFN-γ score [239].

### 12.3. Predictive Markers and Personalized Medicine

Predicting response rates, toxicity, and durability present a major challenge to current mM immunotherapies. The melanoma and immune oncology research communities are investing significant resources to identify predictive biomarkers [242] that would allow treatments to be better optimized for each patient’s therapeutic goals. While predictive response markers are still limited, computation algorithms have been developed to provide individualized predictions of response to pembrolizumab in mM patients with some success [243]. Several independent predictive markers of outcome, including tumor-mutational burden, neoantigen load, and pretreatment CD8+ TIL count, have also been identified across multiple immunotherapies [244,245,246,247]. With the high rates at which mM develops therapy resistance, an investigation into how immunotherapies affect tumor signaling, tumor pathology, and the TME must also be considered.

Identification of tumor neoantigens and predictability of immunogenicity poses another issue. There are over 16,200 distinct class I HLA alleles, each with distinct peptide-binding preferences. Predicting which epitopes will likely be presented by each patient’s APCs is key to the future of immunotherapies such as ACT, OVs, and melanoma vaccines, as this interaction ultimately determines the immunogenicity of a given neoantigen. Some recent progress has been made: The HLAthena model can predict endogenous HLA-binding peptides with >75% accuracy [248]. The Tumor Neoantigen Selection Alliance (TESLA) developed a bioinformatic-informed model of tumor-epitope immunogenicity capable of filtering out 98% of nonimmunogenic peptides with a precision of over 0.70 [249]. However, no tool currently exists that can accurately predict if a specific neoantigen–HLA combination will be recognized by an individual’s TCRs. More informed models will require a larger and more diverse data set. The accessibility and affordability of next-generation molecular and functional diagnostics may one day allow each patient to receive personalized immunotherapy, optimized specifically to their tumor and goals.

## 13. Conclusions

While current therapeutic regimens demonstrate clear efficacy in many patients with advanced melanoma, some patients either relapse or do not respond to these regimens. Thus, the development of alternative strategies is still necessary.

We have summarized the current status of the melanoma treatment landscape and outlined results from past and ongoing clinical trials. Optimization of current therapies through rational combinations and optimized selection of alternative drug targets are the next frontier in melanoma treatment, with the ultimate overarching goal of sustained efficacy in the context of a more personalized treatment plan.

## Abbreviations

BRAF	B-Raf proto-oncogene
CSD	Cumulative sun damage
CTL	Cytotoxic T lymphocyte
CTLA-4	Cytotoxic T lymphocyte-associated antigen 4
DC	Dendritic cell
FDA	Food and Drug Administration
HD	High dose
ICAM	Intercellular adhesion molecule
ICI	Immune-checkpoint inhibitors
IFN	Interferon
IL	Interleukin
irAE	Immune-related adverse effect
IV	Intravenous
MAPK	Mitogen-activated protein kinase
MHC	Major histocompatibility complex
mM	Metastatic melanoma
NK	Natural killer (cells)
ORR	Overall response rate
OS	Overall survival
OV	Oncolytic virus
Peg-IFN	Peginterferon
PD-L1	Programmed cell death protein 1 ligand
PD-1	Programmed cell death protein 1
PFS	Progression-free survival
RFS	Relapse-free survival
RR	Response rate
RTK	Receptor tyrosine kinase
SHP-1	Src homology region 2 domain-containing phosphatase-1
SHP-2	Src homology region 2 domain-containing phosphatase-2
Treg	T-regulatory cell
TIL	Tumor-infiltrating lymphocytes
TME	Tumor microenvironment
